# Enhanced thermal conductivity in a hydrated salt PCM system with reduced graphene oxide aqueous dispersion

**DOI:** 10.1039/c7ra10632g

**Published:** 2018-01-03

**Authors:** Xinxing Zhang, Xiang Li, Yuan Zhou, Chunxi Hai, Yue Shen, Xiufeng Ren, Jinbo Zeng

**Affiliations:** Key Laboratory of Comprehensive and Highly Efficient Utilization of Salt Lake Resources, Qinghai Institute of Salt Lakes, Chinese Academy of Sciences Xining 810008 China lixiang@isl.ac.cn zhouy@isl.ac.cn +86 971 6338403 +86 971 6338403; University of Chinese Academy of Sciences Beijing 100049 China; Key Laboratory of Salt Lake Resources Chemistry of Qinghai Province Xining 810008 China

## Abstract

The phase change enthalpy, thermal conductivity, thermal stability and thermal reliability of a novel reduced graphene oxide (r-GO) containing phase change material (PCM) r-GO/CaCl_2_·6H_2_O were investigated. The material was made by the aqueous dispersion of r-GO and calcium chloride dihydrate (CaCl_2_·2H_2_O) according to the mass ratio of CaCl_2_ and crystal water in CaCl_2_·6H_2_O. The thermal conductivity of the phase change material increased by ∼80% when using ∼0.018% (by weight) of r-GO with a ∼2.7% decrease of enthalpy (*i.e.*, storage capacity), while using ∼0.018% of graphite led to an increase of thermal conductivity by ∼14% and a decrease of enthalpy by ∼5.6%. Additionally, the surface active agent for dispersing r-GO had the extra function of enhancing the system stability and reliability. The decomposing temperatures of r-GO/CaCl_2_·6H_2_O were higher than those of CaCl_2_·6H_2_O. After 100 cycles, the melting and crystallizing enthalpies of r-GO/CaCl_2_·6H_2_O decreased to 178.4 J g^−1^ and 150.7 J g^−1^ from 180.6 J g^−1^ and 153.7 J g^−1^, dropping by 1.2% and 2.0%, respectively, while for CaCl_2_·6H_2_O they decreased to 178.9 J g^−1^ and 147.8 J g^−1^ from 185.6 J g^−1^ and 161.8 J g^−1^, dropping by 3.7% and 8.7%, respectively. The thermal conductivity enhancement of CaCl_2_·6H_2_O with r-GO was markedly superior compared to that with graphite and other thermal conductive additives reported in previous literature, and the provided method (*i.e.*, preparing aqueous dispersions of additives firstly and synthesizing hydrated salt PCMs with corresponding salts subsequently) was also applicable for other functional additives that cannot be directly dispersed well to modify the thermal properties of hydrated salt PCM systems.

## Abstract 2

We prepared r-GO/CaCl_2_·6H_2_O by r-GO aqueous dispersion to enhance the thermal conductivity of a hydrated salt PCM.

## Graphical abstract

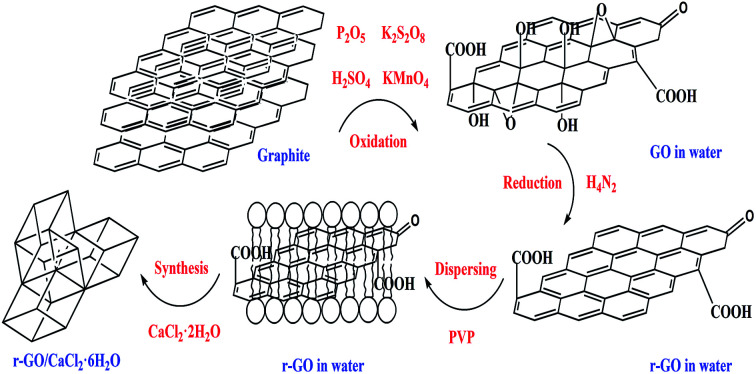

## 1. Introduction

Thermal energy is one of the major forms of energy in the universe, and exists in sources such as solar radiation, natural geothermal energy and oceans in the natural environment. Additionally, it is released as waste heat in the production and operation systems of human society, from artificial facilities such as metal smelting systems, heating systems of buildings and various machines. There is great interest in the development of highly efficient transfer and conversion systems as well as materials to transfer the energy or convert it into other forms of energy such as electricity.^1,2^ Phase change materials (PCMs), possessing the ability to absorb and release energy in the process of changing phase, have drawn a lot of attention due to their high latent heat in applications.^3–6^ One important type is the hydrated salt PCM, which has been a study hotspot in the field of low temperature due to distinct advantages such as high heat fusion, low price, and high security in energy storage systems.^7^ They can be utilized as thermal functional materials for thermal control of buildings,^8^ air conditioning systems^9^ and solar energy.^10^ However, a major defect is their low thermal conductivity which increases the temperature gradients and time constants as well as reduces heat transfer rates. Therefore, modifying the property of thermal conductivity is a critical problem in hydrated salt PCMs.^11,12^ To achieve this goal, many methods have been used including impregnating PCMs into a high thermal conductivity matrix^13^ and adding high conductivity fillers such as oxidation expandable graphite,^14^ nano-copper,^15^ nano-Al_2_O_3_ (ref. 16) and graphite flakes.^17^ Excellent results have been obtained in those works. It is natural that additives with higher thermal conductivity will boost larger thermal conductivity enhancement in PCMs while allowing a smaller quantity of additive to be used, which further leads to a smaller reduction of phase change enthalpy.

Graphene, a honeycomb-structured sheet of carbon atoms, possesses excellent properties including high thermal conductivity. The thermal conductivity of single layer graphene can reach as high as 5300 W m^−1^ K^−1^.^18^ Therefore, it has the potential to be an ideal material for modifying the thermal conductivity of PCMs. However, dispersing graphene directly into hydrated salts is difficult due to its easy agglomeration caused by the Van Edward force between the carbon layers and hydrophobicity.^19^ Graphene oxide (GO) including oxygen-containing functional groups on the surface has a good dispersibility in aqueous dispersions.^20^ However, its thermal conductivity is reduced because of structural defects. To overcome this drawback and keep good dispersivity simultaneously, the method is to reduce the aqueous dispersion of GO to the aqueous dispersion of r-GO which possesses good dispersivity and better thermal conductivity due to the reparation of structural defects.^21,22^ The aqueous dispersion of r-GO with good dispersivity can be utilized directly to synthesize hydrated salts using corresponding salts according to the mass ratio of the salt and crystal water in hydrated salt PCMs. Additionally, it is reported that r-GO can be used to improve thermal conductivity of materials such as polystyrene,^23^ polyimide^24^ and paper.^25^ Therefore, the aqueous dispersion of r-GO could be a promising candidate to enhance the thermal conductivity of hydrated salt PCMs.

Therefore, GO was made using a modified Hummers’ method and further reduced to make a stable r-GO aqueous dispersion using the reducing agent hydrazine hydrate and the surface active agent polyvinyl pyrrolidone (PVP). According to the mass ratio of calcium chloride and crystal water in calcium chloride hexahydrate (CaCl_2_·6H_2_O), calcium chloride dihydrate (CaCl_2_·2H_2_O) was added into the r-GO aqueous dispersion to make the r-GO/CaCl_2_·6H_2_O PCM, and the graphite (G)/CaCl_2_·6H_2_O PCM was also made in order to make a comparison of the boosting effect on the thermal conductivity between r-GO and graphite. A series of thermal properties was tested. It is significant that the improvement of the thermal conductivity should be achieved by sacrificing enthalpy as little as possible. The results in this work show that the r-GO aqueous dispersion causes a large increase in the thermal conductivity of PCMs with a small reduction of enthalpy, which is superior to graphite and other additives such as nano-Al_2_O_3_, nano-Cu and graphite flakes in the previous studies, and the method (*i.e.*, preparing aqueous dispersions of additives firstly and synthesizing hydrated salt PCMs with corresponding salts subsequently) is also a promising way for other additives that cannot be directly dispersed well in hydrated salt PCM systems to modify thermal properties. Additionally, the active agent PVP used for dispersing r-GO has extra functions to enhance the stability and reliability of hydrated salts. In this work, the new system of the r-GO/CaCl_2_·6H_2_O PCM was prepared with r-GO aqueous dispersion to effectively enhance the thermal conductivity by means of firstly preparing aqueous dispersions of additives, and secondly synthesizing hydrated salt PCMs with corresponding salts. Meanwhile, other phase change behaviors such as the supercooling degree, phase change temperature, latent heat, thermal stability and reliability of the PCM in the presence of r-GO were also studied.

## 2. Experimental section

### 2.1 Materials

Graphite powder (C, high dispersivity) was purchased from Yuandong oil refinery company, China. Potassium persulfate (K_2_S_2_O_8_, analytically pure), potassium permanganate (KMnO_4_, guarantee reagent), sulphuric acid (H_2_SO_4_, 98%), hydrochloric acid (HCl, analytically pure), hydrogen peroxide (H_2_O_2_, guaranteed reagent), hydrazine hydrate (N_2_H_4_, analytically pure) and calcium chloride dihydrate (CaCl_2_·2H_2_O, analytically pure) were purchased from Sinopharm Chemical Reagent company, China. Phosphorus pentoxide (P_2_O_5_, analytically pure) was purchased from China pharmaceutical company, China. Polyvinyl pyrrolidone (average mol wt 10 000) was supplied by Sigma-Aldrich company, America.

### 2.2 Preparation of additives, aqueous dispersions and PCMs

GO was made from graphite powder following a modified Hummers’ method.^26,27^ Firstly, K_2_S_2_O_8_ (8 g), P_2_O_5_ (8 g) and H_2_SO_4_ (25 mL, 98%) were mixed well before C (5 g) was added into a round bottom flask. Subsequently, the mixture was reacted in a water bath (80 °C) for 5 h. The reaction liquid was diluted to 500 mL with deionized water and left to set for 12 h. The sediment and liquid components were separated, and the sediment was dried at 45 °C for 2 days. Secondly, the sediment (2 g) was added into H_2_SO_4_ (150 mL) in a water bath (0 °C), then KMnO_4_ (25 g) was added to the reaction which was placed in the water bath (35 °C) for 4 h before deionized water (250 mL) was added. When the temperature of the mixture fell below 50 °C, H_2_O_2_ (30 mL, 30%) was added to the mixture to stop the reaction after deionized water (1 L) was added. The obtained mixture was left to set for 2 h and solid GO was obtained through filtration. Further purification methods were used including washing the solid twice with deionized water (1 L) after it was washed with HCl (1 L, 4%), and being further dialyzed for 3–5 days. Finally, the GO sample was used to prepare a 2 mg mL^−1^ aqueous dispersion by means of ultrasound for 2 h.

**Fig. 1:**
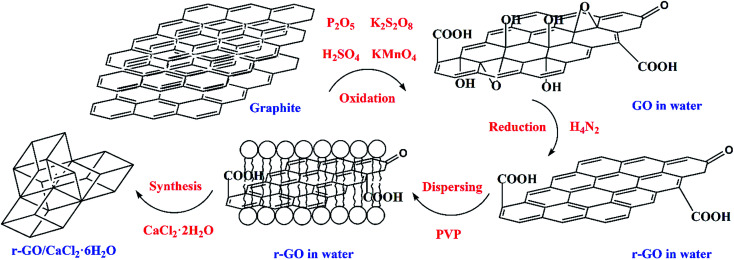
The preparation principle of the r-GO/CaCl_2_·6H_2_O PCM.

Hydrazine hydrate (*M*_1_, 0.4 g, N_2_H_4_, 54.4%) and PVP (*M*_2_, 4.0 g) were added into the GO aqueous dispersion (20 mL) and stirred for 30 min. The dispersion mixture then went through an isothermal reaction in a reactor at 90 °C for 2 h, and the obtained r-GO aqueous dispersion was diluted to 70 mL (3.5 fold dilution) and sonicated for 30 min. The weight of the mixture (*M*_3_) was measured using a high precision balance. Then the weight of the water (*M*_4_) in the r-GO aqueous dispersion was calculated using the formula *M*_4_ = *M*_3_ − *M*_1_ × 54.4% − *M*_2_. Finally, according to the stoichiometric ratio of calcium chloride and crystal water in CaCl_2_·6H_2_O, CaCl_2_·2H_2_O (2.045*M*_4_) was added into the aqueous dispersion of r-GO to prepare CaCl_2_·6H_2_O with the first step of stirring for 5 min and the second step of a 30 min ultrasound. The principle of the preparation of the r-GO/CaCl_2_·6H_2_O PCM is shown in Fig. 1. The average volume and surface area sizes of the graphite (G) particles are 9.103 μm and 4.607 μm, respectively. The G aqueous dispersion (2 mg mL^−1^) was made by ultrasound first, and the G/CaCl_2_·6H_2_O PCM was made by following the same steps as those for the r-GO/CaCl_2_·6H_2_O PCM without the step of the reduction reaction.

### 2.3 Characterization

The particle size distribution was detected by a laser particle size analyzer (Mastersizer 2000). The UV spectra were measured using a UV-Vis spectrophotometer (PerkinElmer Lambda750) with wavenumber ranging from 200 to 800 nm. The Raman spectra were measured by a micro-Raman spectrometer (DXR) with a 532 nm laser. The crystalline phases of the samples were measured using X-ray diffraction (X’PRO Pert). Thermal conductivities were obtained from a thermal conductivity tester (HDRX3A) at 30 °C. The thermal properties were measured by using differential scanning calorimetry (DSC, TA Q20), where the samples were sealed in an alumina pan in a purified nitrogen atmosphere. The viscosity was tested using a Digital Viscometer (NDJ-8S) (testing range: 1–2 × 10^6^ mpa s) with 30 rpm at 22 °C. Thermal stability testing was performed simultaneously using DSC-TGA (Q600), where samples were heated from 25 °C to 500 °C at a rate of 5 °C min^−1^ under a nitrogen atmosphere. The estimated uncertainties are listed in Table 1.

**Table 1:** The estimated uncertainties of the measurements

Property	Estimated uncertainty
Mass	0.12 mg
Temperature (DSC)	0.058–0.58 °C
Latent enthalpy	0.0012
Thermal conductivity	0.058

## 3. Results and discussion

### 3.1 Characterization of GO and r-GO

**Fig. 2:**
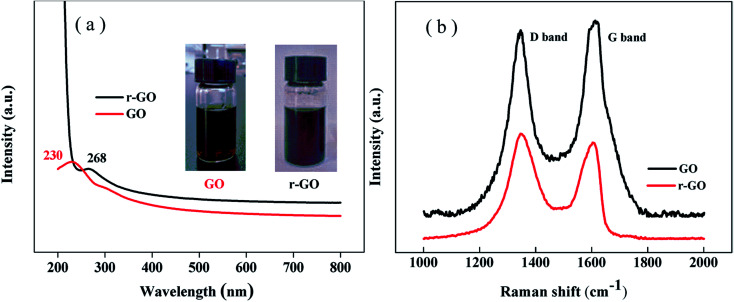
(a) The UV and (b) Raman spectra of GO and r-GO.

The reduction of graphene oxide is characterized by the color changing from brown (GO) to black (r-GO) as shown in the inset of Fig. 2(a). The recovery of the black color in r-GO indicates the re-graphitization of GO due to the removal of oxygen-containing functional groups. Fig. 2(a) shows that the peak in the UV spectrum of GO appears at 230 nm due to the π–π* transition of aromatic C=C bonds,^28^ while the peak is red shifted to 268 nm in the spectrum of r-GO, which is attributed to the enhancement of electron concentration and structural ordering as well as the increased linking model of the sp^2^ carbon atoms.^29^ This characterizes the restoration of the graphene structure in r-GO.

It has been reported that Raman spectroscopy can study disorders and defects in the crystal structures of graphite and its derivatives, and disorder can be investigated using the intensity ratio of the disorder induced D band and the Raman allowed G band (*I*_D_/*I*_G_). In Fig. 2(b), the Raman spectra show that the G band appears at 1612 cm^−1^ and 1605 cm^−1^, whereas the D band is at 1347 cm^−1^ and 1346 cm^−1^ for GO and r-GO, respectively. It has been reported that the G band is caused by the C–C bond stretch of sp^2^ carbon^30^ and originates from the first order Raman scattering.^31^ In this work, the G band of r-GO is shifted to a lower wavenumber because of the increasing number of sp^2^ carbon atoms, and the *I*_D_/*I*_G_ is 1.12 (for r-GO) compared to 0.93 (for GO), which agrees with the previous results,^32^ indicating an increase in conjugated carbon atoms followed by the removal of oxygen containing groups in r-GO. These characterizations prove that the reduction of GO has been achieved by the method in this work.

### 3.2 The dispersing stabilities of additives in aqueous solutions and PCMs

**Fig. 3:**
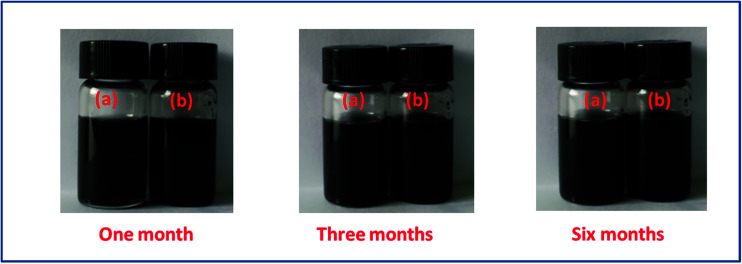
The dispersing stability of (a) the G aqueous dispersion and (b) the r-GO aqueous dispersion.

**Fig. 4:**
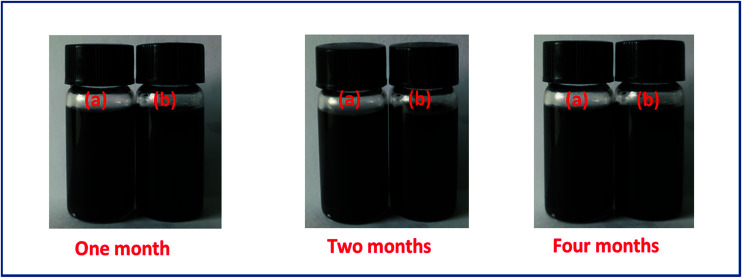
The dispersing stability of the additives in (a) G/CaCl_2_·6H_2_O and (b) r-GO/CaCl_2_·6H_2_O PCMs.

The dispersivity of additives is the key factor to guarantee thermal conductivity enhancement in PCMs. Fig. 3 shows the stability of r-GO and G aqueous dispersions over six months. The result indicates that both of the aqueous dispersions keep good stability in the testing, which is attributed to the function of the surface active agent (PVP). Additionally, Fig. 4 demonstrates that the r-GO in r-GO/CaCl_2_·6H_2_O and the G in G/CaCl_2_·6H_2_O have good dispersivities over a test period of four months. These stable dispersivities of additives can cause good performance on boosting the thermal conductivities of hydrated salt PCMs.

### 3.3 Material identification of PCMs

**Fig. 5:**
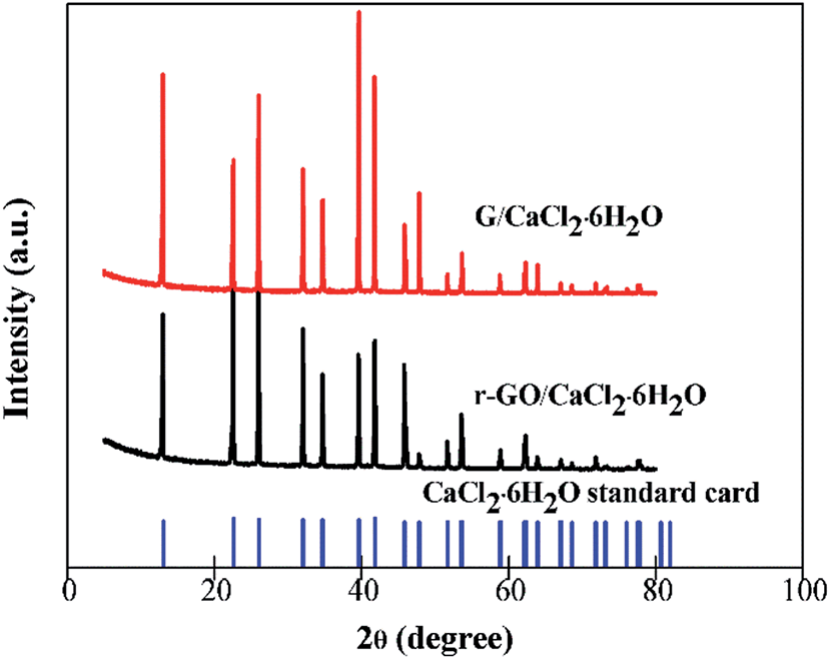
The XRD patterns of G/CaCl_2_·6H_2_O and r-GO/CaCl_2_·6H_2_O PCMs.

CaCl_2_ has two other kinds of hydrated salt form which are CaCl_2_·4H_2_O and CaCl_2_·2H_2_O, and it is necessary to establish the existence of the structure of CaCl_2_·6H_2_O when other components are combined with it to make composite PCMs. The XRD patterns of G/CaCl_2_·6H_2_O, r-GO/CaCl_2_·6H_2_O and the standard card of CaCl_2_·6H_2_O are shown in Fig. 5. All of the characteristic peaks of r-GO/CaCl_2_·6H_2_O and G/CaCl_2_·6H_2_O are consistent with the peaks of the standard card of CaCl_2_·6H_2_O, which illustrates that the method of combination of CaCl_2_·2H_2_O and the aqueous dispersions of additives can make a composite PCM of CaCl_2_·6H_2_O, and the dispersing G, r-GO and PVP do not affect the formation of the crystal phase of CaCl_2_·6H_2_O. This demonstrates the function of G/CaCl_2_·6H_2_O and r-GO/CaCl_2_·6H_2_O as phase change materials in practical applications.

### 3.4 The thermal conductivities of PCMs

**Fig. 6:**
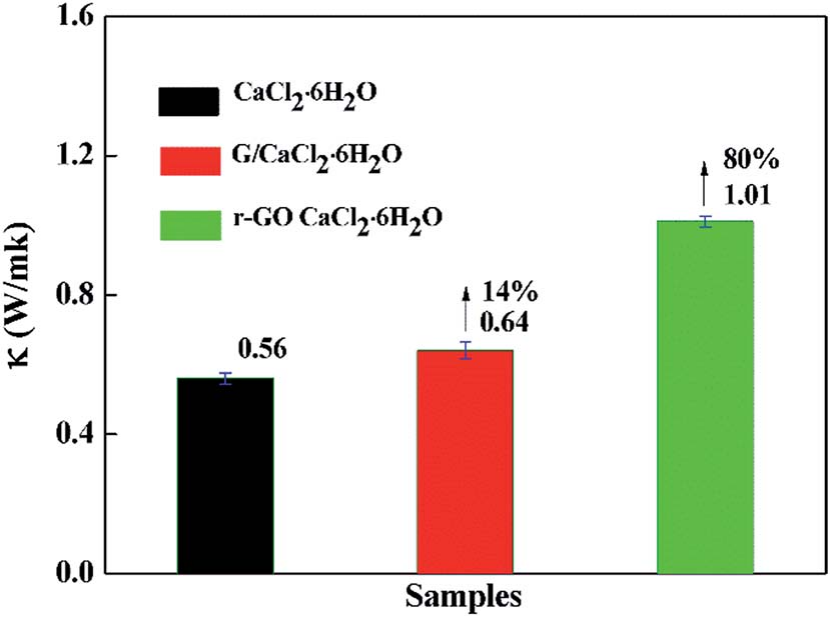
The thermal conductivities of CaCl_2_·6H_2_O, G/CaCl_2_·6H_2_O and r-GO/CaCl_2_·6H_2_O PCMs.

The rate of energy storage and release is an important property in thermal storage systems, and it depends on the thermal conductivity of the phase change materials. The thermal conductivities of samples were measured at 30 °C by the transient hot-wire method and the results are presented in Fig. 6. The thermal conductivity of CaCl_2_·6H_2_O is 0.56 W m^−1^ K^−1^, which is consistent with the reported results,^7,33^ while the thermal conductivities of G/CaCl_2_·6H_2_O and r-GO/CaCl_2_·6H_2_O are 0.64 W m^−1^ K^−1^ and 1.01 W m^−1^ K^−1^, respectively, which proves that G and r-GO can boost the thermal conductivity of hydrated salts and r-GO has a greater effect than graphite due to its layered and reticular structure. Additionally, the large increase for r-GO/CaCl_2_·6H_2_O is caused by the high thermal conductivity of the r-GO network, in which phonons travel with less resistance. Also, the large interfacial contact area and the strong interface between the PCM and r-GO help to improve the thermal conductivity of r-GO/CaCl_2_·6H_2_O.

Theoretically, considering the vast thermal conductivity of graphene, the thermal conductivity of r-GO/CaCl_2_·6H_2_O should give a higher enhancement than the obtained value in this work. However, the structural defects in r-GO reduce the effect of boosting the thermal conductivity of PCMs.^21^ Additionally, the main heat transfer mechanism is attributed to lattice vibrations or phonons in materials, but in prepared r-GO/CaCl_2_·6H_2_O, weak phonon coupling in the vibrational modes at the (CaCl_2_·6H_2_O)–(PVP)–(r-GO) interfaces causes large thermal resistance, also named the kapitza resistance, which reduces the thermal conductivity of r-GO/CaCl_2_·6H_2_O.^34,35^

### 3.5 The thermal properties of PCMs

**Fig. 7:**
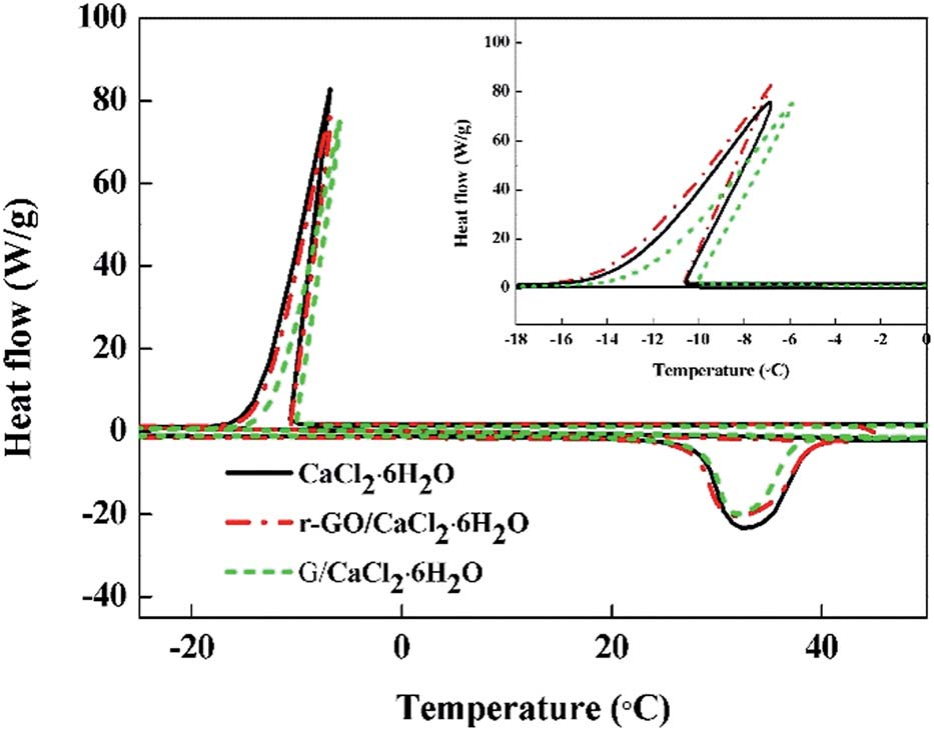
The DSC curves of CaCl_2_·6H_2_O, G/CaCl_2_·6H_2_O and r-GO/CaCl_2_·6H_2_O PCMs.

Phase change enthalpy is a vital property in PCMs and it can be utilized as an index to evaluate the thermal energy storage capacity of PCMs. Therefore, DSC was performed to test the thermal properties of PCMs with the results shown in Fig. 7 and Table 2. As shown in Table 2, the melting enthalpies are 175.2 J g^−1^, 180.6 J g^−1^ and 185.6 J g^−1^ for G/CaCl_2_·6H_2_O, r-GO/CaCl_2_·6H_2_O and CaCl_2_·6H_2_O, respectively. The decreased enthalpies are caused by parts of the PCM being replaced by the additives that do not undergo the phase change, and the larger decrease for G/CaCl_2_·6H_2_O may be caused by the larger size of graphite preventing the crystallization of CaCl_2_·6H_2_O. In previous cases, adding ∼4.5% of Al_2_O_3_ into hydrated salts leads to a ∼61.3% increase in the thermal conductivity and an ∼8% decrease in the enthalpy,^16^ adding ∼0.5% of nano-Cu into hydrated salt leads to a ∼20% increase in the thermal conductivity and a ∼3.3% decrease in the enthalpy,^15^ and adding ∼5% of graphite flakes into hydrated salts leads to a ∼61% increase in the thermal conductivity and at least a ∼5% decrease in the enthalpy.^17^ In this work, adding ∼0.018% graphite leads to a ∼14% increase in the thermal conductivity and a 5.6% decrease in the enthalpy, while adding ∼0.018% r-GO leads to an ∼80% increase in the thermal conductivity and a ∼2.7% decrease in the enthalpy (Fig. 6 and 7). These comparisons with graphite, r-Al_2_O_3_, nano-Cu and graphite flakes show that utilizing the r-GO aqueous dispersion is a more efficient way to enhance the thermal conductivity of hydrated salt PCMs.

**Table 2:** The thermal properties of G/CaCl_2_·6H_2_O, r-GO/CaCl_2_·6H_2_O and CaCl_2_·6H_2_O

Sample	Melting enthalpy (J g^−1^)	Crystallizing enthalpy (J g^−1^)	Melting temperature (°C)	Crystallizing temperature (°C)
CaCl_2_·6H_2_O	185.6	161.8	28.4	−10.5
r-GO/CaCl_2_·6H_2_O	180.6	153.7	27.8	−10.4
G/CaCl_2_·6H_2_O	175.2	140.4	28.5	−10.1

It is also noted that the crystallizing enthalpies of G/CaCl_2_·6H_2_O, r-GO/CaCl_2_·6H_2_O and CaCl_2_·6H_2_O are 140.4 J g^−1^, 153.7 J g^−1^, and 161.8 J g^−1^, respectively, which are lower than the melting enthalpies mentioned above. This may be attributed to the special crystallizing process of CaCl_2_·6H_2_O which has two steps, with the first being formation of CaCl_2_·4H_2_O and the second being peritectic reaction of the mixture. These two steps can also be observed from the phase diagram of CaCl_2_–H_2_O.^36^ The step of formation of CaCl_2_·4H_2_O is a metastable state with a fast change of enthalpy that the DSC instrument may not be sensitive enough to detect, as there is no peak for the first step of crystallization in the DSC curves.^37^

Additionally, the crystallizing temperatures of PCMs do not change significantly, with −10.1 °C, −10.4 °C and −10.5 °C for G/CaCl_2_·6H_2_O, r-GO/CaCl_2_·6H_2_O and CaCl_2_·6H_2_O, respectively. This indicates that the particles have no influence on the crystallizing temperature of hydrated salts, which may be attributed to the sizes and the morphologies of particles used in this work that do not match the crystal type of CaCl_2_·6H_2_O.^38^

### 3.6 Thermal stability of the r-GO/CaCl_2_·6H_2_O PCM

**Fig. 8:**
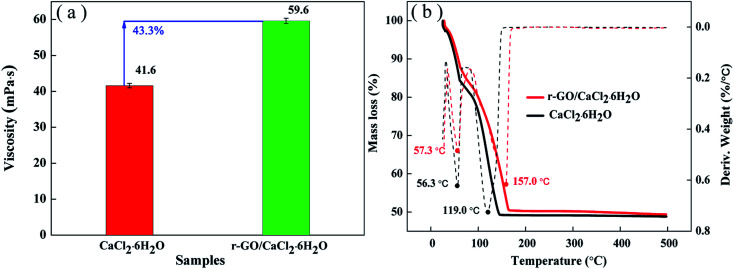
(a) The viscosities and (b) TGA curves of CaCl_2_·6H_2_O and r-GO/CaCl_2_·6H_2_O PCMs.

Thermal stability can be used as a measure to evaluate the adaptive ability of temperature changes in applications. For hydrated salts, the loss of crystal water is a key issue to reduce the heat storage capacity in cycling. The viscosity of the liquid state is an important property to sustain the stability of the system, and high viscosity has the function of inhibiting movement of the water molecules from the liquid system to the environment in the process of a phase change. Therefore, the viscosities of the PCM systems were tested and the results are shown in Fig. 8(a). As shown in Fig. 8(a), the viscosity of r-GO/CaCl_2_·6H_2_O is improved to 59.6 mpa s from 41.6 mpa s (for CaCl_2_·6H_2_O) due to the effect of PVP and the uniform dispersion of r-GO in the PCM system.

Then, TGA analysis was performed to test the stability of the PCM under continuous heating conditions. Fig. 8(b) shows the entire TGA analysis curve of both r-GO/CaCl_2_·6H_2_O and CaCl_2_·6H_2_O. The mass loss ending temperature of r-GO/CaCl_2_·6H_2_O is 167 °C which is higher than that of CaCl_2_·6H_2_O (145 °C), and the remaining mass for both PCMs reaches about 49.3% which is lower than the theoretical mass fraction of CaCl_2_ in CaCl_2_·6H_2_O (50.6%) due to the surface water on the hydrated salt crystal. Additionally, the temperatures of the maximum rate of weight loss of r-GO/CaCl_2_·6H_2_O at different stages are 57.3 °C and 157.0 °C, which are higher than 56.3 °C and 119.0 °C (for CaCl_2_·6H_2_O) as shown by the differential curves of mass loss in Fig. 8(b). These results indicate that the prepared r-GO/CaCl_2_·6H_2_O is a more stable system than CaCl_2_·6H_2_O over changing temperature.

### 3.7 The thermal reliability of r-GO/CaCl_2_·6H_2_O

**Fig. 9:**
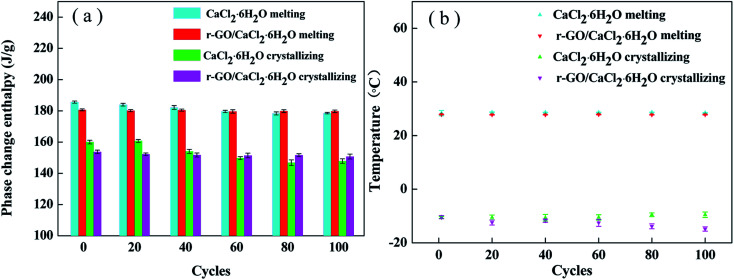
The thermal properties of CaCl_2_·6H_2_O and r-GO/CaCl_2_·6H_2_O after different cycles: (a) the phase change enthalpy and (b) the phase change temperature.

The thermal reliability of PCMs is an essential property for the achievement of economic value, and long operating cycles of PCMs can enhance efficiency and reduce the costs of operation and maintenance of the energy system. Therefore, a test of 100 cycles was conducted using DSC analysis to investigate the thermal reliability of the samples, and the results are shown in Fig. 9. After 100 cycles, the melting and crystallizing enthalpies of r-GO/CaCl_2_·6H_2_O decreased to 178.4 J g^−1^ and 150.7 J g^−1^ from 180.6 J g^−1^ and 153.7 J g^−1^, dropping by 1.2% and 2.0%, respectively, while those of CaCl_2_·6H_2_O decreased to 178.9 J g^−1^ and 147.8 J g^−1^ from 185.6 J g^−1^ and 161.8 J g^−1^, dropping by 3.7% and 8.7%, respectively. Apart from the instrument error, testing errors are also caused by the tiny testing sample size for the DSC instrument.^39^ These data show that the proportions of enthalpy reduction of the r-GO/CaCl_2_·6H_2_O system are lower than those of CaCl_2_·6H_2_O, suggesting that the r-GO/CaCl_2_·6H_2_O system shows better reliability due to the improvement of viscosity. Additionally, the melting temperatures do not show significant changes, and the crystallizing temperatures of r-GO/CaCl_2_·6H_2_O have more widely varying values with a minimum of −14.8 °C and a maximum of −10.4 °C, compared to −10.5 °C and −9.5 °C for CaCl_2_·6H_2_O. This is the main drawback of adding additives, as they change their distribution slightly in the system in long cycles and further influence the susceptible crystallizing temperature. This drawback also increases the testing errors of crystallizing temperatures in the cycling of the r-GO/CaCl_2_·6H_2_O system shown in the error bars. Therefore, further studies are required to solve this problem for adding functional additives.

## 4. Conclusions

It is shown that preparing a hydrated salt PCM system by combining the r-GO aqueous dispersion and the salt is an efficient way to utilize the high thermal conductivity of graphene derivatives. The well-dispersed r-GO has a great effect in boosting the thermal conductivity without causing a large enthalpy reduction in the hydrated salts. ∼0.018% (by weight) of r-GO increases the thermal conductivity by ∼80% and gives a ∼2.7% decrease in enthalpy, while the same amount of graphite incurs a ∼14% increase in thermal conductivity and a ∼5.6% decrease in enthalpy, and the performance of r-GO is also superior to other thermal additives reported in previous literature. Additionally, the well-dispersed r-GO and the surface active agent for dispersing r-GO can help to enhance the stability and reliability of the hydrated salt systems. The decomposing temperatures of r-GO/CaCl_2_·6H_2_O are higher than those of CaCl_2_·6H_2_O. After 100 cycles, the melting enthalpy of r-GO/CaCl_2_·6H_2_O decreases to 178.4 J g^−1^ from 180.6 J g^−1^, only dropping by 1.2%, while that of CaCl_2_·6H_2_O decreases to 178.9 J g^−1^ from 185.6 J g^−1^, dropping by 3.7%. These results indicate that utilizing the r-GO aqueous dispersion has advantages to improve the thermal properties of hydrated salts including thermal conductivity, thermal stability and reliability without a significant reduction of the enthalpy. Additionally, the method used in this work can be applied to other functional additive materials which cannot be directly dispersed well into hydrated salt PCMs, and it is applicable in hydrated salt phase change material systems for the modification of thermal properties.

## Conflicts of interest

The authors have declared no conflicts of interest.

## Supplementary Material
